# Aggregation Behavior of Nano-Silica in Polyvinyl Alcohol/Polyacrylamide Hydrogels Based on Dissipative Particle Dynamics

**DOI:** 10.3390/polym9110611

**Published:** 2017-11-14

**Authors:** Qinghua Wei, Yanen Wang, Yingfeng Zhang, Xiongbiao Chen

**Affiliations:** 1 Department of Industry Engineering, College of Mechanical Engineering, Northwestern Polytechnical University, Xi’an 710072, China; weiqinghua@mail.nwpu.edu.cn (Q.W.); zhangyf@nwpu.edu.cn (Y.Z.); 2 Department of Mechanical Engineering, College of Engineering, University of Saskatchewan, Saskatoon, SK S7N5A9, Canada

**Keywords:** nano-silica, PVA/PAM blended hydrogel, dissipative particle dynamics, aggregation behavior, relative concentration distributions

## Abstract

Due to the aggregation behavior of nano-silica in aqueous solution, the use of nano-silica without surface modification for synthesizing hydrogels is still a challenging task. This paper presents our study on the use of dissipative particle dynamics simulations to discover the aggregation behavior of nano-silica in polyvinyl alcohol (PVA)/polyacrylamide (PAM) blended hydrogels. By simulations, we aimed at investigating the effects of such factors as nano-silica content, polymer component ratio, temperature and shear rate on the aggregation behavior of nano-silica in terms of the mesoscopic morphologies and the relative concentration distribution functions. Our results reveal that the dispersion of nano-silica is seen if the nano-silica content is increased to 1.5%, and the aggregation of nano-silica becomes noticeable in blended hydrogels with an increase in the nano-silica content. This finding agrees well with the experimental results obtained by means of scanning electron microscopy. Furthermore, it is also found that the dispersion of nano-silica becomes more uniform with an increase in PAM content, temperature and shear rate. These findings greatly enrich our understanding of the aggregation behavior of nano-silica in PVA/PAM blended hydrogels.

## 1. Introduction

Polymer blending has become more important than ever in the synthesis of homopolymers and copolymers over the last decade, and allows for creating new materials with properties appropriate for many applications at low cost [1,2,3]. This is also true in tissue engineering [4,5,6]. With their good biocompatibility, biological activity and three-dimensional network structure, polyvinyl alcohol (PVA) and polyacrylamide (PAM) have been widely used and blended in the preparation of biomedical hydrogels [7,8]. Due to their poor mechanical properties [8,9], however, the application of PVA/PAM blended hydrogels has been limited in applications such as bone and cartilage repair, where the mechanical properties are of critical importance [10,11,12,13]. Recent studies have shown that the introduction of nano-silica particles into polymeric materials can not only endow polymer scaffolds with biomineralization capability, but also increase the mechanical strength of polymer material [14,15]. Silica derivatives have been introduced as bone substitutes [16] to promote new vital bone around these materials [17], and as bio-mimetic agents to coat implant surfaces for improvement [18]. Additionally, nano-silica processed in biomaterials also assists with osteoblast cell proliferation [19,20,21]. Thus, adding nano-silica particles into a PVA/PAM blended composite can not only improve mechanical properties, but also promote proliferation of cells in the blended hydrogel. These advantages make the PVA/PAM/nano-silica blended hydrogel suitable for bone-tissue engineering. However, due to the large number of hydroxyl groups existing on the surface of nano-silica [22], the nano-silica tends to aggregate together in the blended hydrogel. This aggregation behavior of nano-silica would easily cause stress concentration and greatly weaken the mechanical properties of the blended hydrogel. Therefore, how to control the nano-silica dispersed uniformly in blended hydrogels is the key challenge for the preparation of PVA/PAM/nano-silica blended hydrogels. At present, one of the most common approaches to hinder the aggregation of nano-silica is surface modification [23,24], but this approach usually has to introduce some new materials to the blended hydrogel, which may be harmful for cells or may degrade the other properties of the modified hydrogel. Thus, understanding the aggregation behavior of nano-silica in blended hydrogels is extremely important for the preparation of biomedical hydrogels used in tissue engineering. However, the aggregation of nano-silica in blended hydrogels is a mesoscopic phenomenon, which is hard to discover by experiments and even by molecular dynamics simulations.

To alleviate the above problem, recently, the dissipative particle dynamics (DPD) method has been employed in the literature. DPD is a mesoscopic simulation technique and has been extensively employed in studies of the self-assembly of block copolymers [25], micelles [26], polymer blends [27], surfactants [28] and so on. As an example of application, Gai et al. [29] investigated the phase morphologies of the ultrahigh-molecular-weight polyethylene/polypropylene/poly(ethylene glycol) (UHMWPE) blends, and discovered the effects of shear rates and volume fractions of each of the blended components on the end-to-end distances of UHMWPE, diffusivities and mesoscale morphologies of the blends. Shi et al. [30] employed the dissipative particle dynamics method to investigate the properties of a water/benzene/caprolactam system in the absence or presence of different non-ionic surfactants. Dai et al. [31] investigated the micellization behavior of platycodin at the mesoscopic level by DPD simulations. Also, Chen et al. [32] studied the formation and stabilization of gold nanoparticles in the poly(ethylene oxide)–poly(propylene oxide)–poly(ethyleneoxide) (PEO–PPO–PEO) block copolymer micelle, and their results showed that the formation of gold nanoparticles was controlled by the competition between the aggregation of primary gold clusters and the stabilization by micelles of block copolymers. These studies enriched the understanding of the phase morphology features of the blended systems. However, to our best knowledge, there are no studies reported to discover the aggregation behavior of nano-silica in blended hydrogels with the DPD method. 

In this paper, we present a study on the use of DPD simulations to investigate the effects of silica content, polymer composition, temperature and shear rate on the aggregation behavior of nano-silica in a PVA/PAM blended hydrogel. The influence of these factors and the aggregation behavior of nano-silica were investigated in terms of the phase morphology and relative concentration distribution function of nano-silica. This research will provide insight and guidance for synthesizing nano-silica/polymer blended hydrogels for biomedical applications.

## 2. Modeling and Methods

### 2.1. Dissipative Particle Dynamics Method

In the DPD method, a group of atoms or a volume of fluid, which is large on the atomistic scale but is still macroscopically small, is represented by beads. The bead positions and velocities in DPD are governed by the Newtonian law of motion, that is,

(1) $$\frac{d{\vec{r}}_{i}}{dt}={\vec{v}}_{i},\;m_{i}\frac{d{\vec{v}}_{i}}{dt}={\vec{F}}_{i},$$

where $\vec{r}$, ${\vec{v}}_{i}$, *m_i_* and ${\vec{F}}_{i}$ denote the position vector, velocity, mass and total force of the *i*th particle, respectively. The total force *F_i_* between each pair of beads contains three parts: a harmonic conservative interaction force (${\vec{F}}_{ij}^{C}$), a dissipative force (${\vec{F}}_{ij}^{D}$) and a random force (${\vec{F}}_{ij}^{R}$). These forces are given by

(2) $${\vec{F}}_{i}=\sum_{i\neq j}\left({\vec{F}}_{ij}^{C}+{\vec{F}}_{ij}^{D}+{\vec{F}}_{ij}^{R}\right),$$

(3) $${\vec{F}}_{ij}^{C}=\{\begin{array}{ll} a_{ij}(1-r_{ij}){\vec{e}}_{ij} & \left(r_{ij}<1\right)\\ 0 & \left(r_{ij}>1\right) \end{array},$$

(4) $${\vec{F}}_{ij}^{D}=-\eta \omega^{D}(r_{ij})({\vec{e}}_{ij}\cdot {\vec{v}}_{ij}){\vec{e}}_{ij},\;\mathrm{and}$$

(5) $${\vec{F}}_{ij}^{R}=\sigma \omega^{R}(r_{ij})\xi_{ij}\frac{1}{\sqrt{\Delta t}}{\vec{e}}_{ij},$$

where $r_{ij}=\left|{\vec{r}}_{ij}\right|=\left|{\vec{r}}_{i}-{\vec{r}}_{j}\right|$, ${\vec{e}}_{ij}={\vec{r}}_{ij}/r_{ij}$, ${\vec{v}}_{ij}={\vec{v}}_{i}-{\vec{v}}_{j}$; *η* is the dissipation strength; *σ* is the noise strength; $\omega^{D}(r_{ij})$ and $\omega^{R}(r_{ij})$ represent r-dependent weight functions for the dissipative and random forces, respectively; $\xi_{ij}$ denotes a randomly fluctuating variable with 0 mean and unit variance; Δt is the time step of simulation; and $a_{ij}$ is a constant to describe the maximum repulsion between interacting beads. From the fluctuation–dissipation theorem [33], one has the following equations:

(6) $$\omega^{D}(r)={\left[\omega^{R}(r)\right]}^{2},$$

(7) $$\sigma^{2}=2\eta k_{B}T,\;\mathrm{and}$$

(8) $${\left[\omega^{R}(r_{ij})\right]}^{2}=\{\begin{array}{ll} {\left(1-\frac{r_{ij}}{r_{c}}\right)}^{2} & \left(r_{ij}<r_{c}\right)\\ 0 & \left(r_{ij}\geq r_{c}\right) \end{array},$$

where *k_B_* is the Boltzmann constant, *T* is the temperature in Kelvin and *r_c_* is the cut-off radius. In DPD simulations, the bead mass *m* and the cutoff radius *r_c_* are chosen as the unit of mass and unit of length, respectively. The thermal energy *k_B_T* at the room temperature is chosen as the unit of energy (*E_ref_*), where *T* is the absolute temperature and *k_B_* is the Boltzmann constant. A DPD time unit is the amount of time required for a bead to diffuse its own radius under thermal fluctuations. Therefore, the time scale depends upon the size of the bead, that is, $\tau =\sqrt{mr_{c}^{2}/k_{B}T}$. This ensures all the physical quantities used in the DPD simulation are dimensionless. In addition, a spring force acts between beads which are connected in molecules, and it can be described as [34]:

(9) $${\vec{F}}_{i}^{S}=\sum_{j}C{\vec{r}}_{ij},$$

where *C* is the spring constant and set as 4.0 in the present work.

### 2.2. Models and Interaction Parameters

**Figure 1:**
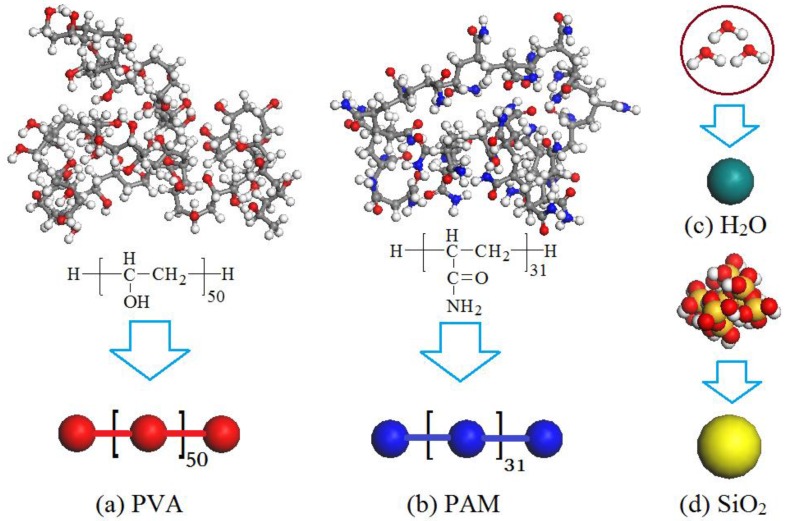
Chemical structures and coarse-grained models of (**a**) PVA: polyvinyl alcohol; (**b**) PAM: polyacrylamide; (**c**) water; and (**d**) SiO_2_.

The DPD simulation is a mesoscopic approach that relies on the construction of a coarse-grained model. An unsuitably coarse-grained model would result in a large deviation of the simulation results [35], so the construction of the coarse-grained model is vitally important for a DPD simulation. In our work, there were four types of beads. Each repeat unit of PVA and PAM were represented as a red bead (Figure 1a) and a blue bead (Figure 1b), three water molecules accumulated as a dark-green bead (Figure 1c) and a molecule of silica was represented as a yellow bead (Figure 1d). Based on some of our previous research [36,37,38,39], the repeat unit numbers of PVA and PAM were set as 50 and 31, respectively. Figure 1 shows the chemical structures and coarse-grained models used in our simulation.

In the DPD simulation, the repulsion parameter ($a_{ii}$) for the same type of beads can be obtained by [40]:

(10) $$a_{ii}=\frac{75k_{B}T}{\rho },$$

where *ρ* is the density of beads with a typical value set as 3, *k_B_* is the Boltzmann constant and *T* is the absolute temperature. In this study, we set the values of *k_B_T* as 1.0, 1.1 and 1.2, corresponding to the temperatures of 298 K, 328 K and 358 K, respectively.

For the different types of beads, the repulsion parameter $a_{ij}$ between different types of beads is related to the Flory–Huggins parameter $\chi_{ij}$ linearly, given by [33]:

(11) $$a_{ij}=a_{ii}+3.27\chi_{ij},$$

where $\chi_{ij}$ is the Flory–Huggins parameter and calculated from the averaged mixing energies, that is,

(12) $$\chi_{ij}=z\left[\frac{E_{ij}-1/2(E_{ii}+E_{jj})}{RT}\right],$$

where *z* is the coordination number of each pair of fragments; *E_ij_* is the mix energy of component *i* and *j*; *R* is the gas constant; and *T* is temperature. In addition, the COMPASS force field was chosen to calculate the Flory–Huggins parameter of each pair of beads. The calculated $\chi_{ij}$ and $a_{ij}$ parameters at different temperatures are given in Table 1.

All the DPD simulations were performed with the Materials Studio 5.5 software (Accelrys, San Diego, CA, USA). The simulation box was set as 15 × 15 × 15 *r_c_*^3^, containing a total of 10,125 representative beads (*ρ* = 3) with periodic boundary conditions under the canonical ensemble (NVT). For the dissipative forces, *η* was set to *η* = 4.5, and for the random forces, *σ* was set to *σ* = 3.00, 3.15 and 3.29 at 298, 328 and 358 K, respectively. In all DPD simulation models of blended hydrogels, the sum occupied volume of polymer beads was set as 20% with the rest occupied by nano-silica and water beads; these beads were uniformly distributed in the initial simulation box. It should be noted that in DPD simulations, all beads have the same volume, where the length unit *r_c_* (also the bead diameter) has a value of 6.46 Å. The time unit *τ* can be calculated by using $\tau =(14.1\pm 0.1)N_{m}^{5/3}$ (ps), as per the work by Groot and Rabone [42], and with $\alpha_{ii}=25$, the calculated time unit is τ≈93.1 ps. Thus, time steps of 0.01, 0.03, 0.05 and 0.07 correspond, respectively, to DPD simulations of 0.931, 2.793, 4.655 and 6.517 ps.

## 3. Results and Discussion

### 3.1. Parametiric Study on the Time Step Used in Simulations

**Figure 2:**
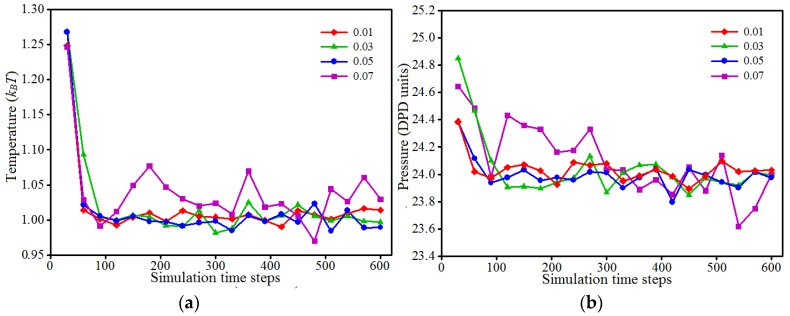
Simulated temperature (**a**) and pressure (**b**) for the 10% PVA/10% PAM/2% nano-silica blended hydrogel system with different time steps; DPD: dissipative particle dynamics.

In simulations, the time step, or the amount of time to update the bead positions and momenta, is critical and its value must be chosen appropriately by trading off simulation accuracy and simulation time. Generally, the smaller the time step is, the more accurate the results are; however, a smaller time step might lead to a significant time amount needed for simulation. In contrast, a larger time step may lead to the problems associated with simulation accuracy and even simulation stability [43]. In our research, we performed a parametric study on the time step by means of four values of 0.01, 0.03, 0.05 and 0.07 ns. Figure 2 shows the difference in both temperature and pressure fluctuations for the 10% PVA/10% PAM/2% nano-silica blended hydrogel system. The results show some slight differences in both temperature and pressure if the time step is set within 0.01–0.05 ns, but large differences if the time step is 0.07 ns as compared to other values within 0.01–0.05 ns. Based on these results, along with the consideration of the simulation time, we chose the time step as 0.05 ns, with a total 50,000 steps towards to the equilibration phase, in our simulations as presented below.

### 3.2. Dynamics Process of the Aggregation of Nano-Silica and Equilibrium

**Figure 3:**
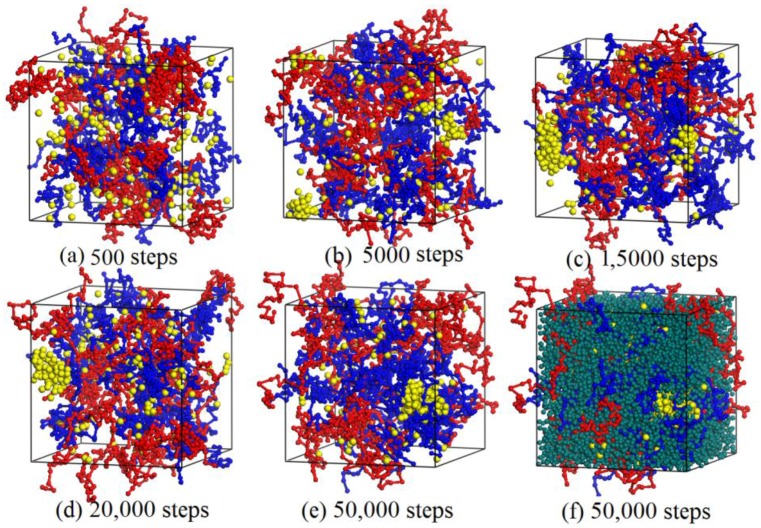
Dynamics process of the aggregation of nano-silica in the 10% PVA/10% PAM/2% nano-silica blended hydrogel system: (**a**) 500 steps; (**b**) 5000 steps; (**c**) 15,000 steps; (**d**) 20,000 steps; (**e**) 50,000 steps; and (**f**) equilibrium model with all beads shown at 50,000 steps. The red, blue and yellow denote PVA, PAM and nano-silica, respectively; and for the rest, dark green denotes water.

**Figure 4:**
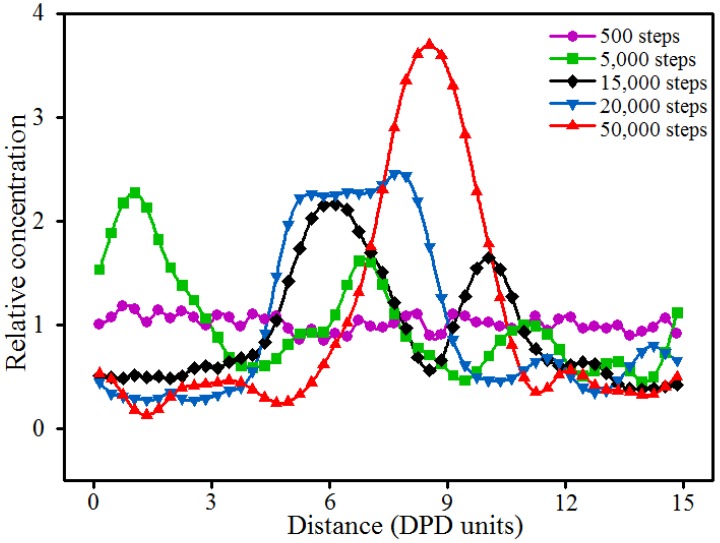
Relative concentration distribution functions of nano-silica in the 10% PVA/10% PAM/2% nano-silica blended hydrogel at different simulation time steps.

Representation of the dynamics process of the aggregation of nano-silica in the blended hydrogel by the DPD simulation method is of importance for researchers to deeply study the change of phase morphologies and the aggregation behavior of nano-silica. In this study, the blended hydrogel model of 10% PVA/10% PAM/2% nano-silica (the component ratio of PVA/PAM/nano-silica is 10%:10%:2%) was selected to study the dynamics process of the aggregation of nano-silica. Figure 3 shows the dynamics process of the aggregation of nano-silica in the 10% PVA/10% PAM/2% nano-silica blended hydrogel system. To clearly show the morphologies of the polymer and nano-silica, all the water beads in the blended hydrogel system are not shown, except in Figure 3f. Figure 4 shows the relative concentration distribution functions of nano-silica in the blended hydrogel corresponding to the morphologies in Figure 3. Here, the relative concentration is given by the ratio of concentration of a type of bead in the slab to its average concentration across the entire system, that is,

(13) $$\rho_{relative}=\frac{N_{slab}/V_{slab}}{N_{total}/V_{total}},$$

where *N_slab_* and *N_slab_* are the numbers of a type of bead in the slab and entire system, and *V_slab_* and *V_total_* are the volumes of the slab and entire system, respectively. Thus, the relative concentration of a homogeneous structure has a value close to 1.0.

As shown in Figure 3, before the DPD simulation, the nano-silica beads were dispersed randomly in the PVA/PAM blended hydrogel (Figure 3a). With the simulation proceeding, the nano-silica start to aggregate together, and the size of the agglomerated particles increases; meanwhile, the discrete nano-silica beads in the blended hydrogel decrease (Figure 3b–e). To further detail the aggregation behavior of nano-silica, the relative concentration distribution functions of nano-silica in the blended hydrogel were analyzed (Figure 4). By comparing the number and magnitude of the peak values existing in the relative concentration distribution functions, the number and size of nano-silica agglomerated particles can be evalauted. The more peaks, the larger the number of nano-silica agglomerated particles present in the blended hydrogel. Similarly, a larger peak value suggests a bigger nano-silica agglomerated particle. The results in Figure 4 indicate that with the increase of simulation time, the size of the agglomerated nano-silica particles increases and the number decreases, which is consistent with the morphologies shown in Figure 3. 

**Figure 5:**
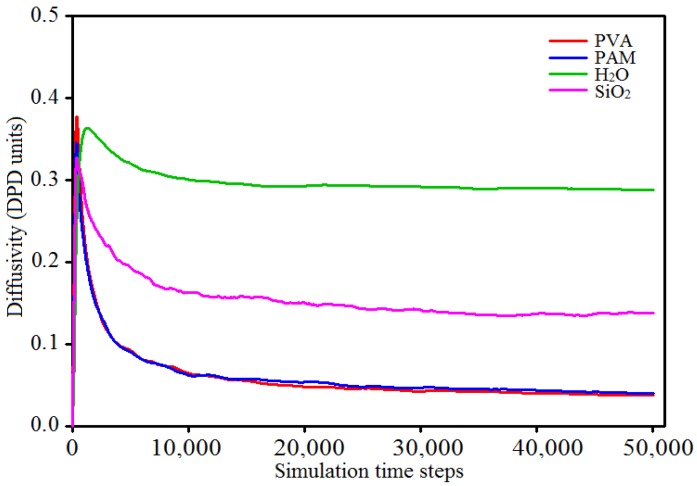
Curves of bead diffusion coefficients in the 10% PVA/10% PAM/2% nano-silica blended hydrogel system versus the simulation time steps.

For simulations, an equilibrium calculation model, which determines the accuracy and reliability of the analysis results, is of vital importance. In DPD simulations, the diffusion coefficients of different beads can be used to determine the equilibrium. Specifically, when the diffusion coefficient of beads converges to a constant, the simulation system is considered to reach equilibrium. The diffusion coefficients D of the beads can be calculated by the Einstein relationship [44]:

(14) $$D=\frac{1}{6N}\underset{t\to \infty }{\lim}\frac{d}{dt}<{\left|r_{i}(t)-r_{i}(0)\right|}^{2}>,$$

where *r_i_*(0) is the initial positional coordinate of bead *i*, *r_i_*(*t*) denotes the coordinates at the time of *t*, and *N* is the number of diffusion beads in the blended systems. Figure 5 shows the diffusion coefficients of beads in the 10% PVA/10% PAM/2% nano-silica blended hydrogel system converging to the constant values after 30,000 simulation time steps (1500 DPD units), which indicates that the total simulation time steps of 50,000 (2500 DPD units) is enough for the blended hydrogel system to reach equilibrium in our work.

### 3.3. Effect of Nano-Silica Content

**Figure 6:**
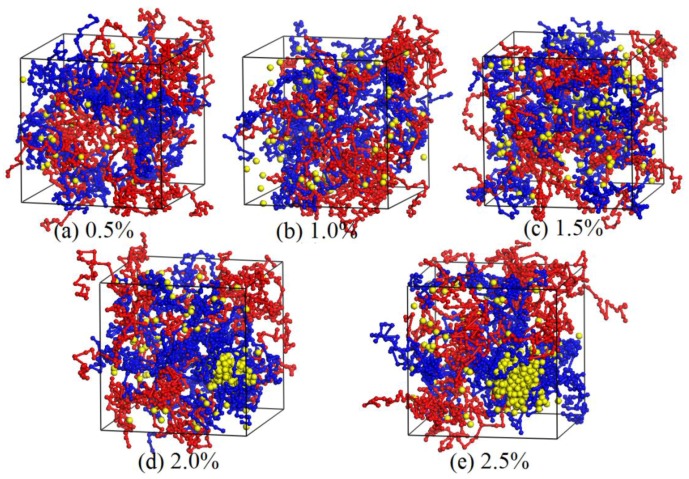
Equilibrated morphologies of 10% PVA/10% PAM blended hydrogels with different nano-silica contents: (**a**) 0.5%; (**b**) 1.0%; (**c**) 1.5%; (**d**) 2.0%; and (**e**) 2.5%.

**Figure 7:**
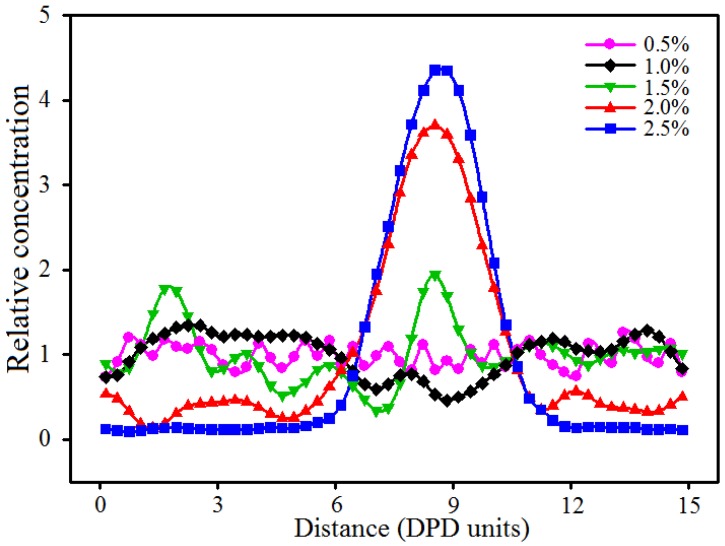
Relative concentration distribution functions of nano-silica in 10% PVA/10% PAM blended hydrogels with different nano-silica contents.

As discussed above, the mechanical properties of blended hydrogels increase with the addition of nano-silica if the nano-silica can disperse uniformly in the hydrogel. However, if the nano-silica cannot disperse uniformly in the hydrogel, the aggregation behavior usually results in concentration stress, which greatly weakens the original mechanical properties of the blended hydrogel. Thereby, the appropriate concentration of nano-silica is important for the preparation of PVA/PAM/nano-silica blended hydrogels. To determine the effect of nano-silica content on the aggregation of nano-silica in the blended hydrogel, a number of PVA/PAM/nano-silica blended hydrogels with different nano-silica contents (0.0%, 0.5%, 1.0%, 1.5%, 2.0% and 2.5%) were constructed. In addition, the contents of both PVA and PAM were 10% in each of the DPD models. Figure 6 and Figure 7 show the equilibrated morphologies and the relative concentration distribution functions of nano-silica in the 10% PVA/10% PAM blended hydrogels with different nano-silica contents at a temperature of 298 K.

From the equilibrated morphologies in Figure 6 and the relative concentration distribution functions of nano-silica in Figure 7, it can be seen that, if the nano-silica content is 0.5% (Figure 6a) and 1.0% (Figure 6b), the nano-silica disperses uniformly in the blended hydrogels without any aggregation phenomenon. In other words, the nano-silica can disperse uniformly in 10% PVA/10% PAM blended hydrogels while the nano-silica content is not greater than 1.0%. When the nano-silica content is increased to 1.5%, some small-size agglomerated particles appear in the blended hydrogel (Figure 6c), and most of the nano-silica beads are still discrete in the blended hydrogel. That suggests a nano-silica content of 1.5% in a 10% PVA/10% PAM blended hydrogel is still acceptable. However, as the content of nano-silica is increased to 2.0% (Figure 6d), the aggregation phenomenon can be observed, with only a small part of the nano-silica beads discrete in the 10% PVA/10% PAM blended hydrogel. This aggregation phenomenon of nano-silica beads becomes more significant as the content is increased to 2.5%. Therefore, to avoid the aggregation of nano-silica in 10% PVA/10% PAM blended hydrogels at 298 K, the nano-silica content should be controlled below 1.5%. 

**Figure 8:**
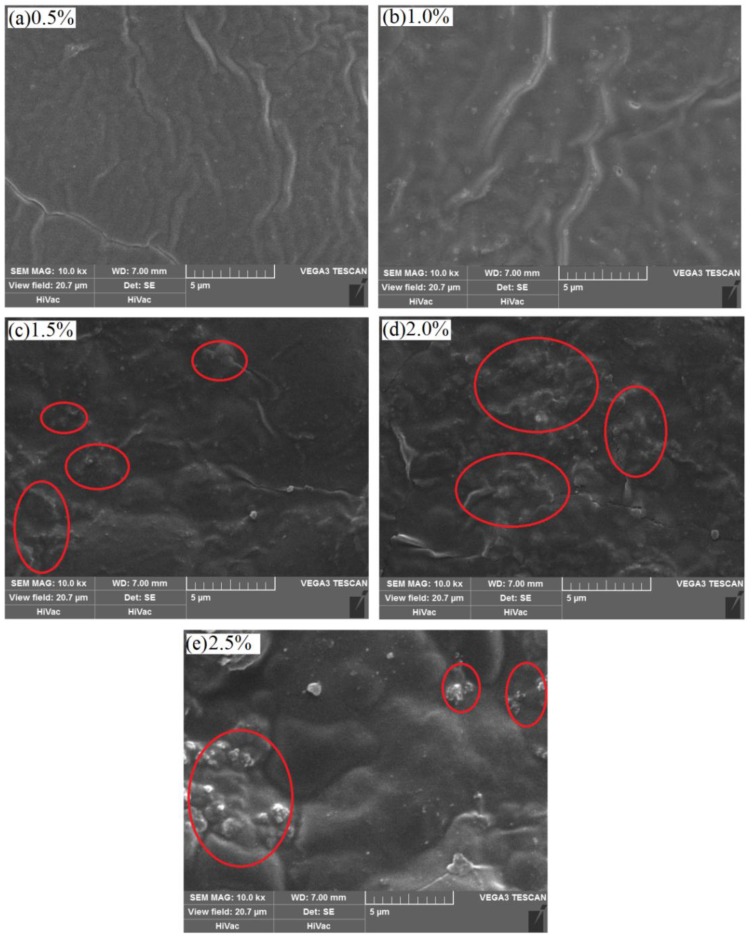
SEM (scanning electron microscopy) images of surface morphologies of 10% PVA/10% PAM blended hydrogels with different nano-silica contents, 10,000× magnification: (**a**) 0.5% nano-silica content; (**b**) 1.0% nano-silica content; (**c**) 1.5% nano-silica content; (**d**) 2.0% nano-silica content; and (**e**) 2.5% nano-silica content.

In order to verify the reliability of our simulations, here, according to the design scheme of DPD models in Figure 6, the PVA/PAM blended hydrogels with different nano-silica contents were prepared by solution-blending and ultraviolet irradiation crosslinking. Then, their surface morphologies were obtained by scanning electron microscopy (SEM). From the SEM images of surface morphologies of 10% PVA/10% PAM blended hydrogels with different nano-silica contents, there was not any obvious aggregation found in the blended hydrogels with nano-silica content of 0.5% and 1.0% (Figure 8a,b), which suggests that the nano-silica disperses evenly in the blended hydrogel. For the case where the nano-silica content is 1.5% (Figure 8c), a slight aggregation phenomenon can be observed in the blended hydrogel. Similarly, when the nano-silica content is above 2.0% (Figure 8d,e), the aggregation phenomenon of nano-silica in the blended hydrogel becomes more obvious. These results are consistent with the simulation ones, and both illustrate that the aggregation phenomenon becomes more and more obvious as the nano-silica content increases. 

### 3.4. Effect of Polymer Component Ratio

**Figure 9:**
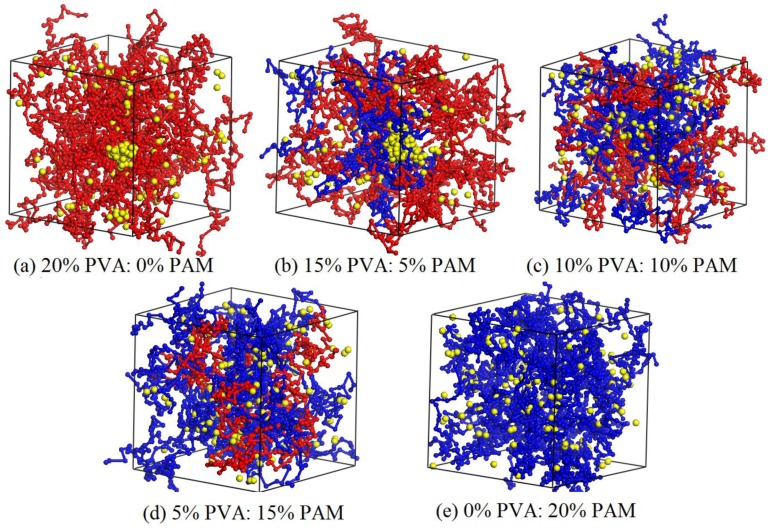
Equilibrated morphologies of PVA/PAM/1.5% nano-silica blended hydrogels with different polymer component ratios at 298 K: (**a**) 20% PVA/0% PAM; (**b**) 15% PVA/5% PAM; (**c**) 10% PVA/10% PAM; (**d**) 5% PVA/15% PAM; and (**e**) 0% PVA/20% PAM.

**Figure 10:**
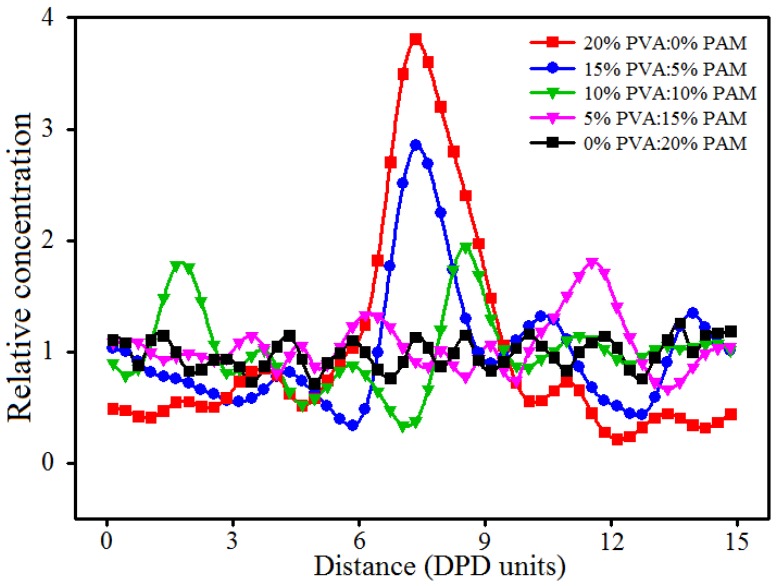
Relative concentration distribution functions of nano-silica in PVA/PAM/1.5% nano-silica blended hydrogels with different polymer component ratios.

Based on the results above, the nano-silica can disperse evenly in the 10% PVA/10% PAM blended hydrogel when its content is not greater than 1.5%. Thus, the nano-silica content of 1.5% was selected to investigate the effect of polymer component ratios on the aggregation behavior of nano-silica. Here, five PVA/PAM/1.5% nano-silica blended hydrogel systems with different polymer component ratios (20% PVA/0% PAM, 15% PVA/5% PAM, 10% PVA/10% PAM, 5% PVA/15% PAM and 0% PVA/20% PAM) were constructed and simulated at a temperature of 298 K. Figure 9 and Figure 10 are the equilibrated morphologies and the relative concentration distribution functions of nano-silica in PVA/PAM/1.5% nano-silica blended hydrogels with different polymer component ratios at 298 K.

**Figure 11:**
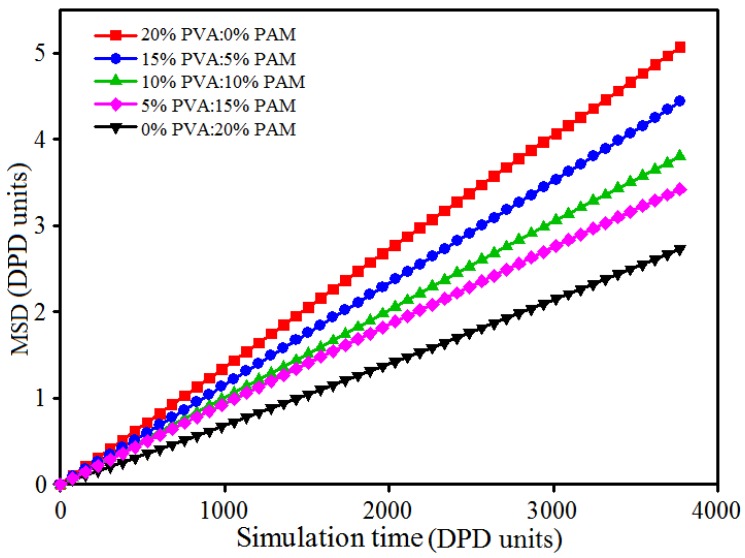
MSD (mean square displacement) curves of nano-silica beads in PVA/PAM/1.5% nano-silica blended hydrogels with different polymer component ratios.

As shown in Figure 9 and Figure 10, the aggregation phenomenon of nano-silica in 20% PVA/0% PAM (pure PVA) hydrogel is the most noticeable, while the dispersion of nano-silica in 0% PVA/20% PAM (pure PAM) is the best. In other words, the dispersion of nano-silica becomes even as the PAM content increases in PVA/PAM blended hydrogels. The most likely reason for this phenomenon is that a stronger interaction force exists between nano-silica and PAM molecular chains compared with PVA molecular chains, and this can also be seen from the interaction parameters of different beads in Table 1. This suggests that the interaction force between nano-silica and polymer in blended hydrogels increases with the PAM content increasing. The increased interaction limits the diffusion of nano-silica and reduces their contact chance in the blended hydrogel system. This explains why the dispersion of nano-silica becomes better with the increase of PAM content in the blended hydrogel. To further support the above conclusion, the mean square displacement (MSD) was used to characterize the diffusion of nano-silica beads. From the curves of MSD for nano-silica beads in Figure 11, one can see that the diffusivity of nano-silica becomes worse with the increase of PAM content in PVA/PAM/1.5% nano-silica blended hydrogels. This indicates that the polymer component ratio greatly affects the diffusion behavior of nano-silica in PVA/PAM/1.5% nano-silica blended hydrogels. In conclusion, the increase of PAM content in blended hydrogels can improve the dispersion of nano-silica in the hydrogel.

### 3.5. Effect of Temperature

**Figure 12:**
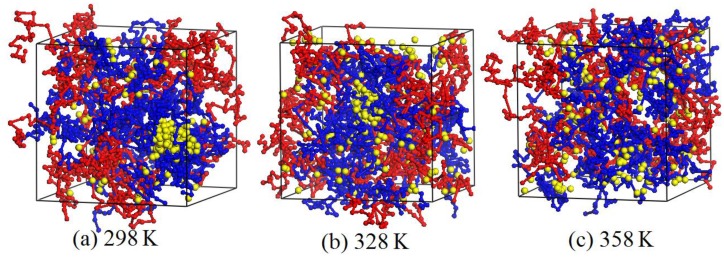
Equilibrated morphologies of 10% PVA/10% PAM/2% nano-silica blended hydrogels at a temperature of (**a**) 298 K; (**b**) 328 K; and (**c**) 358 K.

**Figure 13:**
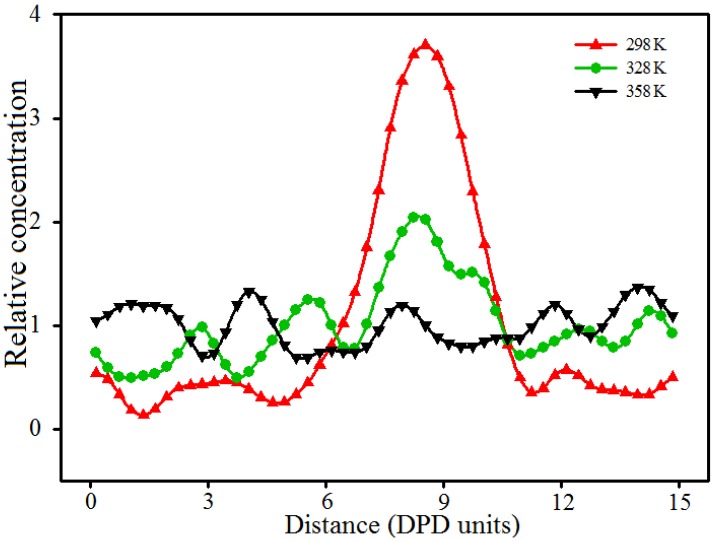
Relative concentration distribution functions of nano-silica in 10% PVA/10% PAM/2% nano-silica blended hydrogels under different temperatures.

During the preparation process of the PVA/PAM/nano-silica blended hydrogels, the temperature shows its effect on the formation and performance of the blended hydrogel, including the dispersion of nano-silica in the system. Thus, knowing the effect of temperature on the dispersion of nano-silica can facilitate the preparation of PVA/PAM/nano-silica blended hydrogels. In our study, the blended hydrogel model of 10% PVA/10% PAM/2% nano-silica was selected to perform the DPD simulations at three temperatures of 298, 328 and 358 K, by using the Flory–Huggins interaction parameters as listed in Table 1. Figure 12 and Figure 13 are the equilibrated morphologies and the relative concentration distribution functions of nano-silica of 10% PVA/10% PAM/2% nano-silica blended hydrogels at the different temperatures.

Figure 12 and Figure 13 illustrate that, if the simulation temperature is 298 K (Figure 12a), the aggregation phenomenon is noticeable and the size of the agglomerated particles in the blended hydrogel is relatively large. When the temperature increases to 328 K (Figure 12b), most of the nano-silica beads still gather together, but the size of the agglomerated particles in the blended hydrogel becomes smaller. However, if the temperature rises to 358 K (Figure 12c), it is hard to notice any agglomerated particles, and the nano-silica disperses evenly in the blended hydrogel. Thus, it is concluded that the dispersion of nano-silica becomes even with an increase of temperature. The main reasons behind this are that the Flory–Huggins interaction parameters and the repulsive interactions between different beads in the blended hydrogel system (Table 1) decrease as the temperature increases, which results in an improved compatibility between different beads. Therefore, within the permitted temperature range of PVA/PAM/nano-silica blended hydrogel preparation, a higher temperature is beneficial for the nano-silica to disperse uniformly in the blended hydrogel. 

### 3.6. Effect of Shear Rate

**Figure 14:**
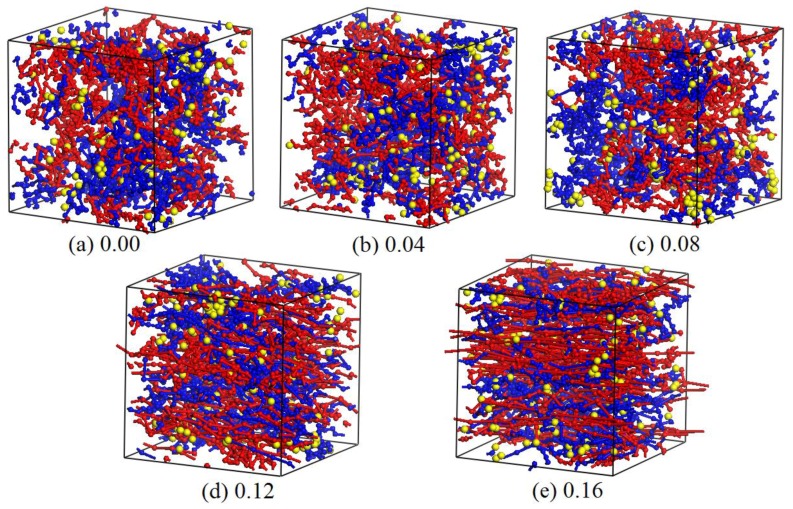
Equilibrated morphologies of 10% PVA/10% PAM/1.5% nano-silica blended hydrogels under different shear rates of (**a**) 0.00; (**b**) 0.04; (**c**) 0.08; (**d**) 0.12; and (**e**) 0.16.

**Figure 15:**
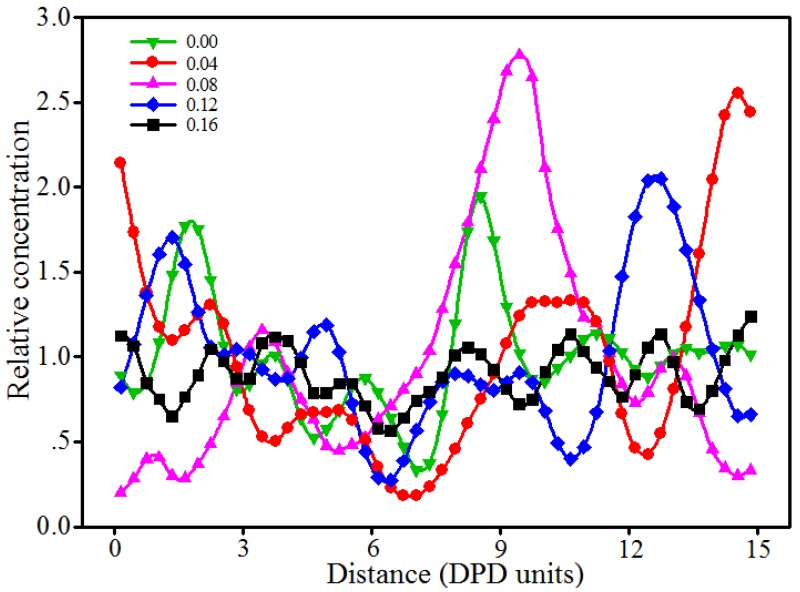
Relative concentration distribution functions of nano-silica in 10% PVA/10% PAM/1.5% nano-silica blended hydrogels under different shear rates.

Shear rate is another factor affecting the phase morphologies of blended hydrogels. This section presents our DPD simulations on the 10% PVA/10% PAM/1.5% nano-silica blended hydrogel system, in which varying levels of shear rates (from 0.00 to 0.16) along the X-axis were applied. The equilibrated morphologies and the relative concentration distribution functions of nano-silica in 10% PVA/10% PAM/1.5% nano-silica blended hydrogels under different shear rates are shown in Figure 14 and Figure 15, respectively.

As seen in Figure 14 and Figure 15, the equilibrated morphologies and the dispersion of nano-silica for the 10% PVA/10% PAM/1.5% nano-silica blended hydrogels are very different, depending on the level of shear rate. At a low shear rate of 0.00–0.08, the aggregation phenomenon of nano-silica in blended hydrogels is noticeable (Figure 14a–c). However, if the shear rate increases to 0.12, the dispersion of nano-silica in blended hydrogels becomes better with the increase of shear rate (Figure 14d,e). With a shear rate of 0.16, the nano-silica almost disperses uniformly in the blended hydrogel (Figure 14e). However, the shear rate can also affect the morphologies of polymers in blended hydrogels, which can be easily observed from the equilibrated morphologies in Figure 14. For the polymers in the blended hydrogel, their morphologies evolve from the original random distribution to the beam distribution, namely, the PVA and PAM molecules in the blended hydrogel are elongated along the shear rate direction and finally show a linear distribution.

**Figure 16:**
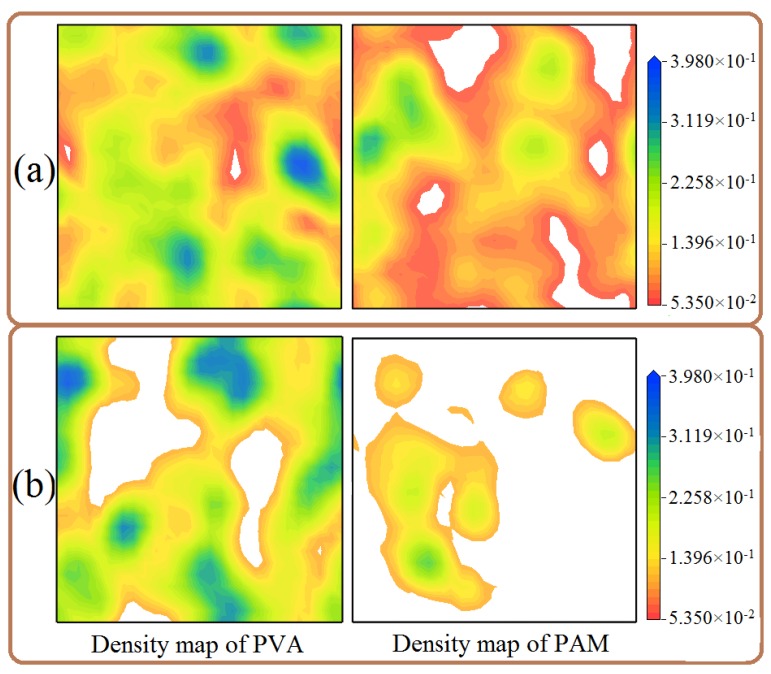
Concentration profile maps of PAM and PVA in 10% PVA/10% PAM/1.5% nano-silica blended hydrogels in the YZ plane with shear rates of (**a**) 0.00 and (**b**) 0.16.

To further investigate the effect of shear rate on the distribution of polymers in the blended hydrogel, two equilibrated morphologies with shear rates of 0.00 and 0.16 were used to analyze the concentration profile maps of PVA and PAM parallel to the XY plane. When the shear rate is 0.00 (Figure 16a), both the concentration profile maps of PVA and PAM cover almost the entire slice, which means their distributions are relatively uniform in the blended hydrogel without the application of shear force. If the shear rate is increased to 0.16 (Figure 16b), the distribution range of PVA and PAM reduces significantly compared to the ones without the application of shear force, especially for the distribution of PAM. Based on the analysis above, it can be concluded the shear rate can affect both the dispersion of nano-silica and the distribution and morphologies of the polymers, which should be taken into account in the preparation of PVA/PAM/nano-silica blended hydrogels.

## 4. Conclusions

In this paper, the DPD simulation method was adopted to investigate the effects of silica content, polymer composition, temperature and shear rate on the aggregation behavior of nano-silica in PVA/PAM blended hydrogels. To discover the aggregation behavior of nano-silica in the hydrogel, different mesoscopic models were designed and analyzed in terms of the equilibrium conformations and the relative concentration distributions of nano-silica in PVA/PAM blended hydrogels. The results reveal that the nano-silica content has a great effect on the aggregation of nano-silica in the blended hydrogel system. The aggregation of nano-silica becomes more obvious with an increase of nano-silica content in the blended hydrogels, and the dispersion of nano-silica is seen even if its content is less than 1.5%. These results agree well with the SEM image results. With an increase of PAM content, the dispersion of nano-silica in the blended hydrogel becomes better, which is attributed to a stronger interaction force between PAM and nano-silica. Furthermore, the dispersion of nano-silica can also be improved by adjusting the temperature for hydrogel preparation, for the reason that the Flory–Huggins interaction parameters and the repulsive interactions between different beads in the blended hydrogel system decrease as the temperature increases, which results in a better compatibility between different beads. Also, our results reveal that the shear rate applied to the hydrogel can affect the aggregation of nano-silica in the blended hydrogel system; specifically, the dispersion of nano-silica can be improved with a shear rate above 0.12. Meanwhile, the distributions and morphologies of the polymers in the blended hydrogel are also be affected by the shear rate, and the morphologies evolve from the original random distribution to the beam distribution.

The results of our study provide insight and understanding of the aggregation behavior of nano-silica in PVA/PAM blended hydrogel systems. This would greatly help the synthesis of PVA/PAM/nano-silica blended hydrogels used in tissue engineering. 

## Figures and Tables

**Table 1:** Flory–Huggins parameters ($\chi_{ij}$) and repulsion parameters ($a_{ij}$) between beads.

Bead Pair	298 K		328 K		358 K	
	$\chi_{ij}$	$a_{ij}$	$\chi_{ij}$	$a_{ij}$	$\chi_{ij}$	$a_{ij}$
PVA–PVA	0.00	25.00	0.00	27.52	0.00	30.03
PVA–H_2_O	0.23, 0.22 [41]	25.75	0.20	28.17	0.18	30.61
PVA–PAM	0.15	25.49	0.14	27.98	0.12	30.42
PVA–SiO_2_	4.17	38.65	3.62	39.35	3.12	40.23
PAM–PAM	0.00	25.00	0.00	27.52	0.00	30.03
PAM–H_2_O	0.57	26.88	0.49	29.12	0.42	31.41
PAM–SiO_2_	2.48	33.11	2.25	34.88	2.01	36.60
H_2_O–SiO_2_	6.35	45.75	5.39	45.15	4.38	44.36
H_2_O–H_2_O	0.00	25.00	0.00	27.52	0.00	30.03
SiO_2_–SiO_2_	−0.44	23.55	−0.52	26.82	−0.61	28.04
